# Vaping-associated nicotine dependence among children and young people in the United Kingdom: time to act

**DOI:** 10.1177/17579139251317835

**Published:** 2025-05-13

**Authors:** R Isba, L Brennan, J Lunn, L Brewster

**Affiliations:** Lancaster Medical School, Lancaster University, Health Innovation Campus, Sir John Fisher Drive, Lancaster, LA1 4YW, UK; Alder Hey Children’s Hospital, Alder Hey Children’s NHS Foundation Trust, UK; Newcastle University, UK; Newcastle City Council, UK; Lancaster Medical School, Lancaster University, UK; Lancaster Medical School, Lancaster University, UK

## Abstract

The authors for this article represent public health, clinical paediatrics, medical sociology, and psychology, and together present an argument for why those in the UK should be concerned about the rise in vaping and vaping-associated nicotine dependence in those under the age of 18. The piece draws together the latest evidence in this area and calls for dedicated services for those who are nicotine dependent as a result of their vape usage, and who are currently overlooked.


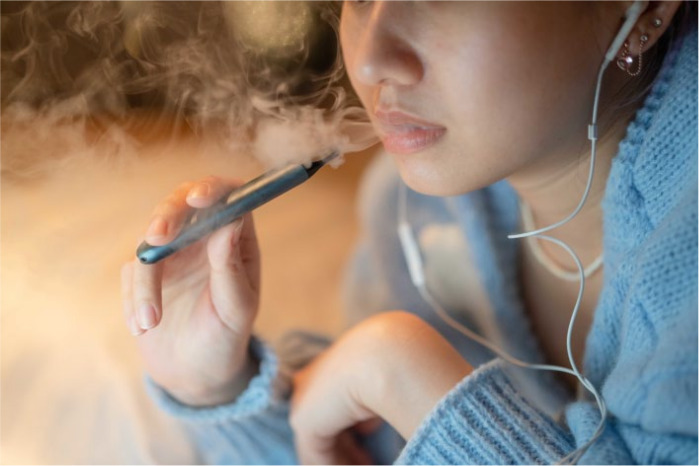


In the United Kingdom, it is illegal to sell vapes (also known as e-cigarettes) to children and young people (CYP) under the age of 18; in the same way, it is illegal to sell them combustible tobacco in the form of cigarettes.

Nevertheless, recent data from Action on Smoking and Health (ASH) suggest that 18% of UK 11- 17-year olds (around 980,000 children) have tried vaping and just over 7% are current vapers, with around 230,000 reporting vaping more than once a week.^1^ Our engagement work with CYP in North West England suggests this may be an underestimate, partly due to CYP being unwilling to disclose vape use to adults.

Available data suggest that the likelihood of vaping increases with age among CYP.^1^ This pattern continues into early adulthood, with recent evidence showing that in England, increasing vaping levels among adults who had never regularly smoked was primarily among 18- 24-year olds.^2^ Although the ASH survey does not include participants under the age of 11, locally collected surveys suggest primary school-aged children are also trying vaping (e.g. Nottinghamshire County Council Public Health^3^).

These concerns formed part of the rationale for the currently paused Tobacco and Vapes Bill, which includes content around youth vaping.^4^ Despite this new law, which seeks to create ‘a smokefree generation and tackle youth vaping’, when it is finally enacted, it is likely to be too late for those CYP already dependent on nicotine as a result of their vaping. While there is a lack of data around the prevalence of this phenomenon, reports suggest ‘. . . there is reason to think vapers are becoming more dependent . . .’, with 44% of regular vapers subjectively describing ‘strong, very strong, or extremely strong’ urges to vape – levels that are comparable with urges to smoke cigarettes among CYP.^1^ A US study found 10.3% of only-vapers aged 11–18 were using their vape within 5 minutes of waking.^5^

Aside from the issue of nicotine dependence, vape use appears to be linked with mental (ill) health^6^ and broader inequalities – for example, CYP who have experienced adverse childhood experiences or have social services involvement are more likely to vape.^7^ Vaping is associated with symptoms such as coughing and wheezing, and side-effects may be more common in those with underlying respiratory conditions such as asthma.^8^ ASH data show that the majority of 11- 17-year olds think that vaping is at least as dangerous as smoking.^1^ Although the dangers of smoking are well-established, the long-term impact of vaping on CYP is unclear and unlikely to be harm free.

The utility of vapes as an effective harm reduction strategy for adults dominates debate around their use,^9^ meaning mixed and confusing messaging exists for CYP around vaping. CYP consistently identify vaping as something they are worried about.^7,10–12^ Several national reports have also called for better data around levels of vaping-associated nicotine dependence in CYP and the development of ‘non-judgemental support’ for those who are addicted to vaping (and smoking), delivered via services which are distinct from those offered to adults.^9,10^ However, the current healthcare landscape in the United Kingdom is almost completely devoid of services to support those under the age of 16 who want to address their vaping. A mismatch between vaping rates among CYP and the number of parents/carers who think that their child vapes may also make it difficult to identify those who need services and may make it more likely that they will come through a ‘punitive’ route such as getting in trouble at school. Where interventions are offered, they are rarely CYP focused.

There are currently no established evidence-based interventions that can be offered in this age group. A 2023 Cochrane review looking at cessation of electronic cigarette use in children and adolescents reported no completed randomised controlled trials (RCTs) that met the criteria for inclusion,^13^ although some studies are currently underway globally. In the United Kingdom, were pharmacotherapy to be used as part of an intervention (as it is in the United States), nicotine replacement therapy (NRT) would need to be prescribed off-licence for anyone under the age of 12, restricting who could deliver services to this younger age group.

Worrying trends including the presence of synthetic cannabinoids such as ‘Spice’ in vapes confiscated in schools, the sale of ‘illicit’ vapes, and the fact that ‘given’ was a very common source of vapes for 11- 17-year olds who participated in the ASH survey, further complicate things. Vaping does not occur in isolation – it is part of the bigger puzzle currently having a negative impact on CYP and captured by a participant in a local vaping survey in Greater Manchester, ‘Most of us know the risks we just don’t care enough about our lives to do anything about it. If the world around us is visibly screwed and we might not last long, why try? Why care?’.^12^

The co-existence of unmet mental health need in CYP who vape^6^ and the mixed evidence of the relationships between vaping and smoking, and vaping and other drug use, mean that any services developed will need to be holistic and inclusive, and accompanied by clear messaging that vaping is not safe (i.e. risk-free) in adolescence. The case for co-development and co-production of services and interventions is strong, as is the argument for dedicated services, staffed by those with experience working with this age group.

High levels of vaping, confusion around health effects, unknown levels of nicotine dependence, a lack of services, an absence of evidence-based interventions, and perhaps most importantly, a consistent call for help from CYP, mean that action needs to be taken now to avoid further harm. This action must take place in the context of wider work around vaping, including the co-development of interventions to prevent vaping among non-vapers, addressing the mixed messages around the safety of vaping, the passing of the Tobacco and Vapes Bill, and the introduction of appropriate support for those CYP for whom the Bill is too late.

## References

[1] Action on Smoking Health. Use of vapes (e-cigarettes) among young people in Great Britain. August 2024. Available online at: https://ash.org.uk/resources/view/use-of-e-cigarettes-among-young-people-in-great-britain (last accessed 28 October 2024).

[2] Jackson SE Shahab L Tattan-Birch H , et al. Vaping among adults in England who have never regularly smoked: a population-based study, 2016-24. Lancet Public Health 2024;9(10):e755–e765. 10.1016/S2468-2667(24)00183-X 39366731

[3] Nottinghamshire County Council Public Health. Vaping in Nottinghamshire – latest information, July 2024. Available online at: https://padlet.com/kaymassingham/notts-vape-free-schools-resource-pack-v20lldho8fvd4u3r/wish/94PGWn8RrNneQLRV (last accessed 28 October 2024).

[4] Department for Health Social Care Media Team. Creating a smokefree generation and tackling youth vaping: what you need to know, 15 April 2024. Available online at: https://healthmedia.blog.gov.uk/2024/04/15/creating-a-smokefree-generation-and-tackling-youth-vaping-what-you-need-to-know/ (last accessed 26 October 2024).

[5] Glantz S Jeffers A Winickoff JP . Nicotine Addiction and intensity of e-cigarette use by Adolescents in the US, 2014-2021. JAMA Network Open 2022;5(11):e2240671. 10.1001/jamanetworkopen.2022.40671 PMC9641541 36342713

[6] Reynolds CME . A review of systematic reviews on the health effects of e-cigarette use in children and adolescents. Dublin; Belfast: Institute of Public Health, 2024. Available online at: https://www.publichealth.ie/news/report-review-systematic-reviews-health-effects-vaping-children-and-adolescents (last accessed 26 October 2024).

[7] The Children’s Commissioner. The Children’s Commissioner’s response to ‘Youth vaping: call for evidence’. 9 June 2023. Available online at: https://www.childrenscommissioner.gov.uk/resource/the-childrens-commissioners-response-to-youth-vaping-call-for-evidence/ (last accessed 26 October 2024).

[8] DiCicco M Sepich M Beni A , et al. How E-cigarettes and vaping can affect asthma in children and adolescents. Curr Op Allergy Clin Immunol 2022;22(2):86–94. 10.1097/ACI.0000000000000807 35197429

[9] Using e-cigarettes to stop smoking. Available online at: https://www.nhs.uk/live-well/quit-smoking/using-e-cigarettes-to-stop-smoking/ (last accessed 26 October 2024).

[10] Public Health Wales. Vaping amongst children and young people in Wales, Incident Response Group, Incident Report, 18 April 2024. Available online at: https://phw.nhs.wales/news/tackle-dependency-visibility-and-availability-to-address-rapid-rise-in-youth-vaping-say-public-health-experts/ (last accessed 26 October 2024).

[11] Amato MS Bottcher MM Cha S , et al. ‘It’s really addictive and I’m trapped’: A qualitative analysis of the reasons for quitting vaping among treatment-seeking young people. Addict Behav 2021;112:106599. 32950927 10.1016/j.addbeh.2020.106599

[12] Healthwatch Trafford. The vaping habits of children and young people in Trafford, July 2024. Available online at: https://nds.healthwatch.co.uk/reports-library/vaping-habits-children-and-young-people-trafford

[13] Barnes C Turon H McCrabb S , et al. Interventions to prevent or cease electronic cigarette use in children and adolescents. Cochrane Database of Systematic Reviews 2023;11:CD015511. 10.1002/14651858.CD015511.pub2 PMC10646968 37965949

